# Knowledge, Attitudes, and Practices of COVID-19 Safety Measures Among Type 1 Diabetes Mellitus Patients at King Abdulaziz University Hospital: A Cross-Sectional Study

**DOI:** 10.7759/cureus.27713

**Published:** 2022-08-05

**Authors:** Abdulaziz A Subyani, Hamza A Fadel, Anas Bokhary, Ahmed M Ghunaim, Hassan H Alharbi, Khalid A Alghamdi, Khalid Alshali

**Affiliations:** 1 Pediatrics, Faculty of Medicine, King Abdulaziz University, Jeddah, SAU; 2 College of Medicine, Faculty of Medicine, King Abdulaziz University, Jeddah, SAU; 3 Internal Medicine, Faculty of Medicine, King Abdulaziz University, Jeddah, SAU; 4 Endocrinology, Faculty of Medicine, King Abdulaziz University, Jeddah, SAU

**Keywords:** type 1 diabetes mellitus, diabetes mellitus, saudi arabia, practice, knowledge, covid-19

## Abstract

Background: Coronavirus disease 2019 (COVID-19), an infectious disease caused by an enveloped RNA beta coronavirus, has become a pandemic, with a mortality rate ranging from 0.7% to 10.8%. Although older adults with comorbidity are more likely to suffer severe disease and eventual mortality, diabetes mellitus (DM) is one of the most frequently reported noncommunicable diseases shown to predict poor prognosis in COVID-19 patients.

Aim: To determine the knowledge, attitudes, and practices (KAP) of COVID-19 safety measures and factors associated with poor knowledge and practice among type 1 diabetes mellitus (T1DM) patients at King Abdulaziz University Hospital (KAUH), Jeddah.

Methodology: This cross-sectional study included 267 patients with T1DM aged 18-65, admitted to the hospital from January to June 2020. Data were collected via phone interviews during September 2020. The questionnaire consisted of 15 questions for knowledge, five for attitude, and eight concerning practices, and some questions were based on diabetes and COVID-19 specifically.

Results: Overall, 64 participants with a median age of 53 years were studied. The median COVID-19 knowledge score showed poor (11.50%), average (43.75%), and good knowledge (6.25%). Most of the participants practiced precautionary measures outside their homes.

Conclusion: In conclusion, half of the population had poor COVID-19 knowledge, 60.9% of the participants felt anxious, and most of them performed precautionary measures, including wearing a facemask, maintaining a 1-m distance, and washing their hands regularly.

## Introduction

Coronavirus disease 2019 (COVID-19), an infectious disease caused by an enveloped RNA beta coronavirus, has become a pandemic [1]. The most common clinical manifestations are fever, fatigue, cough, myalgia, and dyspnea. In contrast, the less common manifestations are sputum production, headache, diarrhea, and hemoptysis [2]. The primary mode of transmission of the virus is via respiratory droplets and contact routes [3]. The incubation period is two to 14 days with a median of five days [4]. On March 11, 2020, COVID-19 outbreak was declared a global pandemic by WHO [5]. More than 564 million people worldwide have been infected with the novel coronavirus according to the latest WHO reports on July 21, 2022 [6]. In Saudi Arabia, the total confirmed cases of COVID-19 were 806,877 as of July 21, 2022 [7].

The mortality rate ranges from 0.7% to 10.8% [8], although older adults with comorbidity are more likely to suffer severe disease and eventual mortality [9]. Diabetes mellitus (DM) is one of the most frequently reported noncommunicable diseases shown to predict poor prognosis in COVID-19 patients [9]. With the lack of a definite treatment for COVID-19, patients with diabetes should consider extra precautions, encourage receiving the vaccine, and ensure good glycemic control [10-12].

Type 1 diabetes mellitus (T1DM) is an autoimmune condition characterized by insulin deficiency caused by the destruction of beta cells in the pancreas [13]. A cure is not available; however, the patients are dependent on long-lasting insulin injections and continuous monitoring of glucose levels [14]. Most T1DM patients tend to have complications, increasing their morbidity and mortality [14,15]. Globally, approximately 463 million adults (aged 20-79 years) lived with diabetes in 2019, of which T1DM accounted for 5% to 10% [16,17]. In accordance with the International Diabetes Federation (IDF), Saudi Arabia is roughly calculated as the fourth highest incidence rate of T1DM in the world, which is higher than any country outside Europe, that is, 31.4 new cases per 100,000 per year [18].

A higher mortality rate was seen in patients with T1DM and COVID-19 in comparison to patients infected with COVID-19 but without T1DM based on a French study [19]. Moreover, glycemic control at the time of infection may also play a role in outcomes. One UK study reporting on deaths in 463 individuals (56.6% male) with COVID-19 and type 1 diabetes suggested a higher mortality risk in those with a hemoglobin A1C (HbA1c) >10% [20].

Previous studies with similar contagious diseases, such as influenza H1N1 flu outbreak, have shown that knowledge, attitudes, and practices (KAP) influence behaviors during a pandemic and play a significant role in personal experience [21]. KAP are crucial adherence factors for a flourishing accomplishment, prevention, and control measures for COVID-19 [22,23]. Moreover, knowledge and attitudes have a more significant impact on emotions, panic, stress, personality, and anxiety [24].

The majority of the participants in a cross-cultural study show an average level of knowledge score upon different territories. However, in the Middle East, the knowledge score mean was comparatively better [25]. A study conducted in India targeting young adults with T1DM showed that most participants had average knowledge and good practice toward COVID-19 [26], whereas participants in a study from Pakistan were noncompliant with the practice of preventive and precautionary measures against the pandemic, despite their knowledge and awareness about the disease [27]. However, in Saudi Arabia, studies have targeted either the general population or health professionals but not the high-risk populations, such as patients with diabetes. Thus, this study aimed to determine the KAP of COVID-19 safety measures and the associated factors of poor knowledge and practice among T1DM patients at the King Abdulaziz University Hospital (KAUH), Jeddah.

## Materials and methods

Data and setting

This cross-sectional study was carried out at KAUH, a tertiary governmental hospital in Jeddah, Saudi Arabia. A total of 267 files were reviewed for patients who were admitted to any of the hospital departments from January to June 2020. All the patients had T1DM and comprised both sexes, aged 18-65 years. Then we excluded patients who refused to participate in the study (88 patients), were deceased (33 patients), or had difficulty establishing contact (due to language barrier, hospital admission, invalid numbers (47 patients), or did not respond to any of the three attempts to reach him/her through phone calls (35 patients)). The research was approved by the King Abdulaziz University Hospital (KAUH) Research Ethics Committee (Reference number: 425-20).

Data collection

We obtained the patients’ electronic data (demographics and phone numbers) from the hospital records using the International Classification of Diseases (ICD)-10 code E10, which refers to insulin-dependent diabetes mellitus. Due to the social distancing measures and the patients’ safety, data were collected through phone interviews conducted, after clarifying the study’s aim to the participants and obtaining their verbal consent in September 2020.

The validated questionnaire used in a previous study was divided into three parts: sociodemographic, diabetes treatment-related details, and knowledge-attitudes-practices (KAP) of COVID-19 safety measures [26]. Sociodemographic details included age, gender, highest educational qualification, employment status, family income per month in Riyals (1 Saudi Riyal (SR)=0.27 USD), and marital status. Diabetes and treatment-related details included whether a glucometer is present in one’s home or not, how possible it was to get their insulin prescription from the pharmacy during the nationwide lockdown, hypoglycemic episodes recurrence and frequency (glucometer readings less than 70 mg/dL), and if there is a history of hospital admission caused by hypoglycemic episode since the beginning of the nationwide lockdown.

Twenty-eight questions are included in KAP datasheet, with knowledge (n=15), attitudes (n=5), and practices (n=8) categories, all related to COVID-19 safety measures. Additionally, some questions were specifically based on the association of DM with COVID-19. Categorically, the first four questions were focused on the severity of the condition, followed by six questions that were related to the transmission of the disease, and the final five questions concerned with illness prevention and control expertise.

There were three choices for each question: “True/False/Do not know". A correct answer received one point, whereas an incorrect/unknown response received zero points added together to give each participant a total score of knowledge that varies from 0 to 15. Scores were used to categorize the level of knowledge. A score of less than or equal to 10 equated to poor knowledge, 11-13 was given for average knowledge patients, and patients with good knowledge received a total score of 14 or more.

Five questions were used to gauge the participants' attitudes on COVID-19 safety measures, including their feelings about being worried about COVID-19, and whether COVID-19 is a virus that can be transmitted from person to person or not, in addition to their belief in their ability to defend themselves and defeat COVID-19. Questions about their behavior when going out in public during the lockdown were used to assess respondents' practices: wearing masks when they leave their homes, COVID-19 prevention through self-protection techniques, and self-care for diabetes.

Statistical analysis

The data were collected using Google Forms (Google LLC, Mountain View, California, United States), then cleaned, checked for completeness, coded on an Excel (Microsoft, Microsoft Corporation, Redmond, Washington, United States) spreadsheet, and analyzed by the Statistical Package for Social Sciences (SPSS, International Business Machines Corporation (IBM), Chicago, United States) version 21.0. The Shapiro-Wilk test and Kolmogorov-Smirnov tests were performed to ensure the data normality. The independent variable “age” and the dependent variable “total knowledge score” were confirmed to be nonparametric and are expressed as median interquartile range (IQR). The Spearman correlation coefficient, based on the normality of data, was used to assess correlations between total knowledge scores and continuous demographic variables (age of participants). In addition to that, comparisons of categorical variables (gender, status of employment, marital status, status of education, and grades of knowledge) with age or total knowledge score were made using the Mann-Whitney or Kruskal-Wallis test. Afterward, participants were categorized as having poor knowledge (less than mean-1 SD), average knowledge (equals mean±1 SD), or good knowledge (more than mean+1 SD). A Chi-square test was performed to determine the association between categorical variables, and a p-value <0.05 was considered significant for all statistical tests.

## Results

Overall, 64 patients participated in the study; the median age was 53 years (interquartile range (IQR): 18.5, range: 18-65). Of them, 34 (53.1%) subjects were men, 20 held a high school degree (31.3%), 38 unemployed (59.3%), 35 with a monthly family income of less than 5,000 Saudi Riyals (54.7%), and 52 married (81.3%). Other demographic characteristics are shown in Appendix Table 1. In diabetes and treatment-related cases, nine participants did not use a glucometer at home (14.1%), and 46 (71.9%) had no problems procuring prescribed insulin preparations from a pharmacy. Further, six (9.4%) had seven to 10 hypoglycemic episodes since the beginning of the nationwide lockdown, and seven (10.9%) required hospital admission more than once for the treatment of hypoglycemia. The other features are summarized in Appendix Table 2.

Knowledge

The COVID-19 knowledge questionnaire has an accurate response percentage of 29.7%-95.3% for the 15 questions (Appendix Table 3). The median COVID-19 knowledge score for all participants was 11 (IQR: 3, range: 0-14); males, 10 (IQR: 3.5); and females, 11 (IQR: 2), with no significant relationship between gender and knowledge score (p=0.102, U=390). The total knowledge score showed a nonsignificant, weak, negative correlation with the participants’ age (r=-0.115, p=0.366).

The number of participants with poor, average, and good knowledge was 32 (50%), 28 (43.75%), and four (6.25%), respectively. There was no significant relationship between age and knowledge grade (p=0.361). Other demographics were tested for relationships with knowledge grades and were found to be insignificant. The knowledge scores of all the participants are shown in Appendix Table 3.

Attitude

In case of attitude, 39 participants felt anxious when they thought about COVID-19 (60.9%), 51 thought that being patients with diabetes makes them vulnerable to COVID-19 (79.7%), 27 considered that following routine dietary advice will weaken their immunity and make them vulnerable to COVID-19 (42.2%), and 61 thought that precautionary measures could protect them from COVID-19 (95.3%). Additionally, 59 participants were sure that the lockdown was helpful in controlling COVID-19 (92.2%). However, no significance was found in relationships between attitudes, practices, and demographics.

Practice

A total of 41 (64.1%) participants had left their homes during the lockdown, and all of them wore a facemask while going outside. A medical mask was favored by 37 (90%), while four (10%) used a homemade mask. Twenty (48.8%) participants had left their homes more than 10 times, and 18 (43.9%) left their homes to purchase needles or glucometer strips from a pharmacy. Only five (12.2%) left their homes for a walk in the park during the lockdown. Regarding the management of T1DM, 24 (37.5%) participants did not follow their routine dietary advice, and 10 (15.6%) did not regularly administer their prescribed doses of insulin. Most of the participants performed precautionary measures, including wearing a facemask, maintaining a 1-m distance, and washing their hands regularly outside their homes (Appendix Table 4).

## Discussion

Thus, this study aimed to assess the KAP of T1DM patients with COVID-19. This is the first study to evaluate patients with type 1 diabetes in the context of COVID-19 in Saudi Arabia. The study showed that many of the participants had poor COVID-19 knowledge. Most thought that being diabetes patients would increase the risk of contracting COVID-19. The Saudi Arabian Ministry of Health (MOH) carried out an intensive awareness campaign and communicated masses about COVID-19 via its website, television, and various social media [28]. However, a very high proportion (50%) of the participants had poor COVID-19 knowledge. This proportion is significantly higher than that reported in studies from Ethiopia [29], Kenya [30], and China [31], which reported a low prevalence of poor knowledge. In a previous cross-cultural study and a study conducted in India, a majority of the participants had an average knowledge [25,26], although a rational explanation of this difference could be the difference in participants’ age, as the mean age in this study was higher than that in other studies.

COVID-19 poses a severe threat, and despite vaccinations, preventive measures play an essential role in reducing the spread of the disease and controlling the infection rates. This indicates the necessity of public adherence to preventive and control measures, which might be affected by knowledge, attitudes, and practices (KAP) of individuals. In a study conducted among elderly patients in Brazil, the participants had low levels of knowledge concerning COVID-19 [32], which supports the studies in the US [33], and China [31], which reported poor knowledge among older adults, supporting age causation. Additionally, access to information electronically is less among older adults. A previous study showed that older adults tend to recall less health information, which supports the age causation explanation [34].

Another explanation for the low knowledge grade in this study could be the educational level, similar to that in the Brazilian study [32]. In India, 74% of the participants were educated (graduate and postgraduate); this clarifies their knowledge grades, which were relatively higher than participants in our study. These results are similar to a study conducted in Iran that showed a very high level of knowledge among highly educated participants with an academic degree [35].

In this study, a majority of the participants with poor knowledge were from low-income families. This is supported by previous studies in Malaysia [36] and the United States [33], which indicated that low-income participants had poor knowledge of COVID-19. Additionally, a study in China indicated that good knowledge and appropriate practice were associated with high income [31]. It is shown that low monthly income leads to a sense of inability to change an individual's behavior or condition, and ultimately, inability to execute recommended protective behaviors for COVID-19 [37,38]. Moreover, an increase in revenue enhances the possibility of meeting the needs to protect from COVID-19. For example, it is possible to purchase a facemask and hand sanitizer when there is sufficient income. Additionally, low-income people do not stay at home and, instead, choose to resume their everyday activities to meet their basic needs. With that in mind, the Saudi government has provided and distributed the vaccine equally to every citizen and foreign, with complimentary and accessibility.

In this study, we found that 60.9% of the participants felt anxious about COVID-19. This could encourage them to follow precautionary measures. Most of the participants thought that being patients with diabetes will increase the risk of contracting COVID-19. The Indian study concurred with this finding [26], which could be because high sugar levels weaken the patient’s immunity, and the participants agreed with this fact. A majority of the participants thought that precautionary measures could protect them from COVID-19. Moreover, most of the participants believed that the lockdown helped control COVID-19. This reflects the huge efforts of the Saudi government, such as the intensive awareness campaigns, including communications via its website, television, and various social media to control the highly infectious pandemic and retain normal life.

This study has some limitations. First, the survey included a small number of participants due to its single-center nature and targeted admission of participants during the lockdown; thus, the results may not be generalizable to all the patients with diabetes in Jeddah. Second, the sample size was affected by the exclusion of children and adolescents (aged <18 years), as we wanted to assess the KAP of the patients and not their parents and also the exclusion of participants who had a language barrier. Finally, the measurement of KAP may be imprecise owing to the limited number of items in the questionnaire especially asking about episodes of attacks of diabetic ketoacidosis. Thus, further studies are needed to expand upon and resolve these issues.

## Conclusions

In conclusion, half of the population had poor COVID-19 knowledge, 60.9% of the participants felt anxious, and most of them performed precautionary measures, including wearing a facemask, maintaining a 1-m distance, and washing their hands regularly. Thus, we advise T1DM patients to take all essential steps to avoid contracting COVID-19, including awareness of the illness, the various transmission modes, and self-protection techniques. Moreover, to improve their innate immune response, persons with diabetes should maintain appropriate glucose control.

## Appendices

**Table 1:** Demographic characteristics of participants. SR, Saudi Riyal.

Characteristics		N (%)
Gender	Male	34 (53.1%)
	Female	30 (46.9%)
Educational level	Illiterate	8 (12.5%)
	Intermediate school	17 (26.6%)
	High school	20 (31.3%)
	Graduate	13 (20.3%)
	Postgraduate	6 (9.4%)
Employment status	Student	2 (3.1%)
	Unemployed	38 (59.3%)
	Employed in government sector	11 (17.2%)
	Employed in private sector	9 (14.1%)
	Self-employed	4 (6.3%)
Monthly family income	<5,000 SR	35 (54.7%)
	5,000-10,000 SR	15 (23.4%)
	>10,000 SR	14 (21.9%)
Marital status	Single	7 (10.9%)
	Married	52 (81.3%)
	Widowed	4 (6.3%)
	Divorced	1 (1.6%)

**Table 2:** Management of blood glucose levels during lockdown.

Features		N (%)
Using glucometer at home	Yes	55 (85.9%)
	No	9 (14.1%)
Did you face any difficulty in obtaining insulin from a pharmacy?	Yes	18 (28.1%)
	No	46 (71.9%)
Times of hypoglycemic attacks since lockdown in Saudi	7-10	6 (9.4%)
	4-6	6 (9.4%)
	1-3	17 (26.6%)
	Never	35 (54.7%)
Times of hospitalization due to hypoglycemic attacks during quarantine	Two or more times	7 (10.9%)
	Once	2 (3.1%)
	Never	55 (85.9%)

**Table 3:** Knowledge of study participants on COVID-19.

Question	Answered right
K-1) People who are infected with coronavirus disease have symptoms like tiredness, fever, shortness of breath, and dry cough.	85.9%
K-2) People who are infected with coronavirus have symptoms and feel ill.	29.7%
K-3) All people infected with coronavirus develop serious or life-threatening disease.	50%
K-4) Diabetes mellitus increases the risk of serious disease and even death.	81.3%
K-5) Infected people can spread the illness to others.	92.2%
K-6) Mode of transmission of coronavirus is respiratory droplets.	81.3%
K-7) Respiratory droplets can land on nearby surfaces and objects and can remain alive for a long period of time.	67.2%
K-8) You can get the coronavirus if you touch your nose, face, eyes, or mouth after coming into contact with coronavirus-infected objects or surfaces.	82.8%
K-9) Individuals infected with coronavirus and have no fever cannot spread the disease.	31.3%
K-10) Insulin injection needles do not transmit the coronavirus.	35.9%
K-11) Young people have a high level of immunity and hence do not require coronavirus protection.	60.9%
K-12) Presently, there is no vaccine or treatment available for COVID-19.	34.4%
K-13) Coronavirus can be prevented by washing your hands with soap and water or using an alcohol-based hand rub on a regular basis.	92.2%
K-14) Coronavirus can be prevented by avoiding crowded places and by keeping a minimum distance of 1 m from others.	95.3%
K-15) If you come into contact with a person who has COVID-19, you should self-isolate for at least 14 days and consult your doctor or hospital if you become ill.	81.3%

**Table 4:** Practice of COVID-19 safety measures. *The percentages are only for those who had left home have been considered as a denominator (n=41).

Questions	Responses	
1) Since general lockdown was declared by the authorities in Saudi Arabia, have you left your home?	Yes	41 (64.1%)
	No	23 (35.9%)
If yes, for how many days?	<5 days	13 (31.7%)*
	6-10 days	8 (19.5%)*
	>10 days	20 (48.8%)*
2) While heading outside, did you put on a facemask?	Yes	41 (100%)*
	No	0 (0%)*
If yes, what type of mask did you wear?	Medical	37 (90%)*
	Homemade	4 (10%)*
3) Did you visit a pharmacy to get glucometer strips or insulin?	Yes	18 (43.9%)*
	No	23 (56.1%)*
4) Did you go out to a park or play with friends?	Yes	5 (12.2%)*
	No	36 (87.8%)*
5) Did you try to maintain at least 1 m distance from others?	Yes	40 (97.6%)*
	No	1 (2.4%)*
6) Are you cleaning your hands with water and soap constantly?	Yes	62 (96.9%)
	No	2 (3.1%)
7) Are you compliant with your dietary advice?	Yes	40 (62.5%)
	No	24 (37.5%)
8) Are you compliant with injecting your insulin doses?	Yes	54 (84.4%)
	No	10 (15.6%)

## References

[1] Lu R, Zhao X, Li J (2020). Genomic characterisation and epidemiology of 2019 novel coronavirus: implications for virus origins and receptor binding. Lancet.

[2] Huang C, Wang Y, Li X (2020). Clinical features of patients infected with 2019 novel coronavirus in Wuhan, China. Lancet.

[3] Desai AN, Patel P (2020). Stopping the spread of COVID-19. JAMA.

[4] Linton NM, Kobayashi T, Yang Y (2020). Incubation period and other epidemiological characteristics of 2019 novel coronavirus infections with right truncation: a statistical analysis of publicly available case data. J Clin Med.

[5] (2022). WHO Director-General’s opening remarks at the media briefing on COVID-19-11 March. https://www.who.int/director-general/speeches/detail/who-director-general-s-opening-remarks-at-the-media-briefing-on-covid-19---11-march-2020.

[6] (2022). WHO: Coronavirus disease (COVID-19) pandemic. https://www.who.int/emergencies/diseases/novel-coronavirus-2019.

[7] (2020). COVID 19 dashboard: Saudi Arabia. https://covid19.moh.gov.sa/.

[8] Omer SB, Malani P, Del Rio C (2020). The COVID-19 pandemic in the US: a clinical update. JAMA.

[9] Pal R, Bhadada SK (2020). COVID-19 and non-communicable diseases. Postgrad Med J.

[10] Pal R, Bhansali A (2020). COVID-19, diabetes mellitus and ACE2: the conundrum. Diabetes Res Clin Pract.

[11] Pal R, Bhadada SK (2020). Should anti-diabetic medications be reconsidered amid COVID-19 pandemic?. Diabetes Res Clin Pract.

[12] Banerjee M, Chakraborty S, Pal R (2020). Diabetes self-management amid COVID-19 pandemic. Diabetes Metab Syndr.

[13] Kerner W, Brückel J (2014). Definition, classification and diagnosis of diabetes mellitus. Exp Clin Endocrinol Diabetes.

[14] Katsarou A, Gudbjörnsdottir S, Rawshani A (2017). Type 1 diabetes mellitus. Nat Rev Dis Primers.

[15] Robert AA, Al-Dawish A, Mujammami M, Al Dawish MA (2018). Type 1 diabetes mellitus in Saudi Arabia: a soaring epidemic. Int J Pediatr.

[16] Melmed S, Polonsky KS, Larsen PR, Kronenberg HM (2015). Williams Textbook of Endocrinology E-Book.

[17] (2022). IDF Diabetes Atlas, 9th Edition. https://www.diabetesatlas.org/en/.

[18] (2017). IDF Diabetes Atlas, 8th Edition. https://www.diabetesatlas.org/en/.

[19] Wargny M, Gourdy P, Ludwig L (2020). Type 1 diabetes in people hospitalized for COVID- 19: new insights from the CORONADO study. Diabetes Care.

[20] Holman N, Knighton P, Kar P (2020). Risk factors for COVID-19-related mortality in people with type 1 and type 2 diabetes in England: a population-based cohort study. Lancet Diabetes Endocrinol.

[21] Yap J, Lee VJ, Yau TY, Ng TP, Tor PC (2010). Knowledge, attitudes and practices towards pandemic influenza among cases, close contacts, and healthcare workers in tropical Singapore: a cross-sectional survey. BMC Public Health.

[22] Ajilore K, Atakiti I, Onyenankeya K (2017). College students’ knowledge, attitudes and adherence to public service announcements on Ebola in Nigeria: suggestions for improving future Ebola prevention education programmes. Health Educ J.

[23] Tachfouti N, Slama K, Berraho M, Nejjari C (2012). The impact of knowledge and attitudes on adherence to tuberculosis treatment: a case-control study in a Moroccan region. Pan Afr Med J.

[24] Markel H (1999). Quarantine!: East European Jewish Immigrants and the New York City Epidemics of 1892.

[25] Ali M, Uddin Z, Banik PC (2021). Knowledge, attitude, practice, and fear of COVID-19: an online-based cross-cultural study. Int J Ment Health Addict.

[26] Pal R, Yadav U, Grover S, Saboo B, Verma A, Bhadada SK (2020). Knowledge, attitudes and practices towards COVID-19 among young adults with type 1 diabetes mellitus amid the nationwide lockdown in India: a cross-sectional survey. Diabetes Res Clin Pract.

[27] Ajay K, Hamza I, Deepika K (2020). Knowledge & awareness about COVID-19 and the practice of respiratory hygiene and other preventive measures among patients with diabetes mellitus in Pakistan. Eur Sci J.

[28] (2022). Saudi MOH. https://twitter.com/SaudiMOH.

[29] Kebede Y, Yitayih Y, Birhanu Z, Mekonen S, Ambelu A (2020). Knowledge, perceptions and preventive practices towards COVID-19 early in the outbreak among Jimma university medical center visitors, Southwest Ethiopia. PLoS One.

[30] Austrian K, Pinchoff J, Tidwell JB (2020). COVID-19 related knowledge, attitudes, practices and needs of households in informal settlements in Nairobi, Kenya [PREPRINT].

[31] Zhong BL, Luo W, Li HM, Zhang QQ, Liu XG, Li WT, Li Y (2020). Knowledge, attitudes, and practices towards COVID-19 among Chinese residents during the rapid rise period of the COVID-19 outbreak: a quick online cross-sectional survey. Int J Biol Sci.

[32] de Lima Filho BF, Bessa NP, Fernandes AC, da Silva Patrício ÍF, de Oliveira Alves N, da Costa Cavalcanti FA (2021). Knowledge levels among elderly people with diabetes mellitus concerning COVID-19: an educational intervention via a teleservice. Acta Diabetol.

[33] Wolf MS, Serper M, Opsasnick L (2020). Awareness, attitudes, and actions related to COVID-19 among adults with chronic conditions at the onset of the U.S. outbreak: a cross-sectional survey. Ann Intern Med.

[34] Morales KJ (2021). General Patterns of Aging, Neurogenic, Psychogenic, and Comorbid Etiology in Older Adults. Regent University ProQuest Dissertations Publishing.

[35] Erfani A, Shahriarirad R, Ranjbar K, Mirahmadizadeh A, Moghadami M (2020). Knowledge, attitude and practice toward the novel coronavirus (COVID-19) outbreak: a population-based survey in Iran. Bull World Health Organ.

[36] Ruggles DR, Freyman RL, Oxenham AJ (2014). Influence of musical training on understanding voiced and whispered speech in noise. PLoS One.

[37] Braveman PA, Cubbin C, Egerter S, Williams DR, Pamuk E (2010). Socioeconomic disparities in health in the United States: what the patterns tell us. Am J Public Health.

[38] Park CL, Cho D, Moore PJ (2018). How does education lead to healthier behaviours? Testing the mediational roles of perceived control, health literacy and social support. Psychol Health.

